# Amygdala functional connectivity in female patients with major depressive disorder with and without suicidal ideation

**DOI:** 10.1186/s12991-018-0208-0

**Published:** 2018-09-12

**Authors:** Shengnan Wei, Miao Chang, Ran Zhang, Xiaowei Jiang, Fei Wang, Yanqing Tang

**Affiliations:** 1 Brain Function Research Section, First Affiliated Hospital, China Medical University, Shenyang, Liaoning People’s Republic of China; 2 Department of Radiology, First Affiliated Hospital, China Medical University, Shenyang, Liaoning People’s Republic of China; 3 Department of Psychiatry, First Affiliated Hospital, China Medical University, Shenyang, 110001 Liaoning People’s Republic of China; 4 Department of Geriatric Medicine, First Affiliated Hospital, China Medical University, Shenyang, Liaoning People’s Republic of China

**Keywords:** First episode, Female, Depressive, Suicidal ideation, Amygdala, Functional connectivity

## Abstract

**Background:**

Major depressive disorder (MDD) is a known major risk factor for suicide and is one of the most common mental disorders. Meanwhile, gender differences in suicidal behavior have long been recognized including the finding that women have higher rates of suicidal ideation and/or suicidal behavior than men. The mechanism underlying suicide ideation in female patients with MDD remains poorly understood. The aim of the present study was to examine possible suicidal behavior-related neural circuitry in female MDD.

**Methods:**

In this study, 15 female participants with the first-episode MDD with suicidal ideation and 24 participants with the first-episode MDD without suicidal ideation as well as 39 female participants in a healthy control (HC) group, ranging in age from 18 to 50 years, underwent resting-state functional magnetic resonance imaging. The whole-brain amygdala resting-state functional connectivity (rsFC) was compared among these three groups.

**Results:**

Compared with female participants with the first-episode MDD without suicidal ideation and those in the HC group, female participants with the first-episode MDD with suicidal ideation showed a significant difference in rsFC between the amygdala and precuneus/cuneus (*p* < 0.05, corrected). No significant difference in amygdala–precuneus/cuneus rsFC was observed between female patients with the first-episode MDD without suicidal ideation and the HC group (*p* < 0.05, corrected).

**Conclusions:**

Our findings suggest that suicidal ideation in female patients with the first-episode MDD may be related to an abnormality in amygdala neural circuitry. The abnormality in amygdala–precuneus/cuneus functional connectivity might present the trait feature for suicide in women with the first-episode MDD. The precuneus/cuneus may be an important region related to suicide and require future study.

## Background

Suicide is an important health problem worldwide. WHO (World Health Organization) reported that suicide is the 20th leading cause of death worldwide. Major depressive disorder (MDD) is a known major risk factor for suicide and is one of the most common mental disorders [1], with a lifetime incidence of 17.1% [2]. Chen and Angst et al. reported that around 15% patients with MDD ultimately die by suicide [3, 4]. The recent study reported that 25% adolescent with MDD had a lifetime history of suicide attempt [5]. Meanwhile, gender is also a strong sociodemographic correlate and has been associated strongly with suicidal behavior. Gender differences in suicidal behavior have long been recognized [6], including the finding that women have higher rates of suicidal ideation and nonfatal suicidal behavior than men [7]. Therefore, the studies of women with MDD are important for identifying factors associated with suicidal behavior.

Structural magnetic resonance imaging (MRI) [8–12] and functional MRI [13] studies have demonstrated brain abnormalities in people with MDD who exhibit suicidal behavior. The amygdala is the key brain region involved in emotional and cognitive processing. The amygdala’s role in processing emotional stimuli has been demonstrated in animal and human research [14–16], and it also has been implicated centrally in people with MDD [17–23]. First, the previous studies also have shown that abnormalities of volume and connectivity of amygdala were associated with the mechanisms of suicide in people with MDD [24, 25]. Second, one study also showed an association between amygdala-middle temporal area connectivity and suicidal ideas in suicide attempters [26]. It was found that suicide attempters showed significantly increased resting-state functional connectivity (rsFC) of the left amygdala with the right insula and left superior orbitofrontal area, and those with MDD showed increased rsFC of the right amygdala with the left middle temporal area [26]. However, the previous study also showed a negative result of amygdala morphometric difference in suicidal patients [27] and another study is the lack of correlation between suicidal ideation and amygdala–precuneus RSFC in adolescents [28].

The mechanism underlying suicide in patients with MDD remains poorly understood. There is little study of the relationship between women with first-episode MDD and suicide. In our study, we performed a seed-based analysis of the amygdala rsFC in female patients with first-episode MDD with suicidal ideation and without suicidal ideation, and healthy control (HC) participants, ranging in age from 18 to 50 years. The goal of the present study was to examine the amygdala rsFC of female patients with first-episode MDD with suicidal ideation and without suicidal ideation.

## Methods

### Participants

The study included 39 participants with first-episode MDD and 39 HC participants between the ages of 18 and 50 years. The first-episode MDD participants were recruited from the outpatient clinics at the Department of Psychiatry, First Affiliated Hospital of China Medical University, Shenyang, China. The HC participants were recruited from Shenyang, China, using community advertisement. The presence or absence of Axis I diagnoses was independently determined by two trained psychiatrists using the Structured Clinical Interview for DSM-IV Axis I Disorders (SCID) in participants 18 years old or older.

The first-episode MDD initially was diagnosed in participants with MDD who had not presented with a manic episode within the 1-year follow-up study. These participants did not present with or have a history of other Axis I disorders, including substance abuse or dependence. HC participants did not have any first-degree relatives with Axis I disorders.

Suicidal ideation, defined as thoughts of engaging in behavior intended to end one’s life, has been identified as an important precursor of both attempted and completed suicide [29–31]. In our study, we used the Beck 19-item Scale for Suicide Ideation [32] to assess whether there has the suicidal ideation. According to our previous research of epidemiology of suicide [33], the presence of suicidal ideation was the outcome variable based on the Beck 19-item Scale for Suicide Ideation; we used the item about suicidal ideation found in the Beck 19-item Scale for Suicide Ideation to stratify our patients. The depressed patients divided into groups with suicidal ideation and without suicidal ideation. We obtained symptom measures using the Hamilton Depression Rating Scale (HAMD).

For all three groups, individuals were excluded if any of the following were present: (1) any MRI contraindications; (2) history of head trauma with loss of consciousness for 5 min or more, or any neurological disorder; (3) any concomitant major medical disorder. All participants were right-handed and scanned within 24 h of the initial contact with the research team. All participants provided written informed consent after reading a detailed description of the study. The Institutional Review Board of China Medical University approved the study.

### MRI data acquisition

We acquired data using a GE (Boston, MA) MR Signa HDx 3.0T MRI scanner at the First Affiliated Hospital, China Medical University, Shenyang, China. Head motion was minimized with restraining foam pads. We used a standard head coil for radio-frequency transmission and reception of the nuclear magnetic resonance signal. The participants were asked to keep their eyes closed but remain awake during the scan. We acquired fMRI images using a spin echo planar imaging (EPI) sequence, parallel to the anterior–posterior commissure plane with the following scan parameters: repetition time (TR) = 2000 ms; echo time (TE) = 40 ms; image matrix = 64 × 64; field of view (FOV) = 24 × 24 cm^2^; 35 contiguous slices of 3 mm without gap; scan time = 6 min 40 s (the 6 min 40 s scans included a total of 200 volumes). We acquired a high-resolution structural image using a three-dimensional fast spoiled gradient-echo T1-weighted sequence: TR = 7.1 ms, TE = 3.2 ms, FOV = 24 cm × 24 cm, matrix = 240 × 240, slice thickness = 1.0 mm without gap, and 176 slices.

### Functional connectivity processing

We conducted resting-state fMRI data preprocessing using SPM8 (http://www.fil.ion.ucl.ac.uk/spm/software/spm8) and the Resting-State fMRI Data Analysis Toolkit (REST; http://www.restfmri.net). The first ten images were deleted, and then, the data underwent further preprocessing, which included slice timing correction, head motion correction, spatial normalization, and smoothing. We computed head motion parameters by estimating translation in each direction and angular rotation about each axis for each volume. We excluded data sets if head motion was more than 3 mm maximum displacement in any of the *x-*, *y-*, or *z*-directions or more than 3° of any angular motion throughout the course of the scan. To assess the head motion confound, we compared the mean framewise displacement [34] among the three groups. The results showed no significant differences in head motion when comparing the three groups (*p* = 0.81). We performed spatial normalization using a standard EPI template from the Montreal Neurological Institute (MNI). The voxel size was resampled to 3 × 3 × 3 mm^3^. We performed spatial smoothing with an 8-mm full-width at half maximum Gaussian filter. To remove low-frequency drifts and physiological high-frequency noise, we performed linear detrending and temporal bandpass (0.01–0.08 Hz) filtering. To remove the effects of nuisance covariates, we performed linear regression of head motion parameters, global mean signal, white matter signal, and cerebrospinal fluid signal [35–37].

### Definition of region of interest

The amygdala was selected as the region of interest (ROI). We defined the bilateral amygdala ROI according to the automated anatomical labeling template [38] contained in REST, which had been resampled to 3 × 3 × 3 mm^3^. We averaged the blood oxygen-level-dependent (BOLD) time series of the voxels within the ROI to generate the reference time series for the ROI.

### Functional connectivity and statistical analysis

We analyzed demographic and clinical characteristics using IBM SPSS Statistics for Windows, Version 21.0 (Armonk, New York).

We performed functional connectivity analysis using correlation analysis between the seed bilateral amygdala ROI and a gray matter mask in a voxel-wise manner using Data Processing and Analysis for Brain Imaging software (DPABI; DPABI_V1.2_141101, http://rfmri.org/dpabi). We then transformed the correlation coefficients to *Z* values using the Fisher’s *r*-to-*z* transformation.

We used one-way analysis of variance (ANOVA) to compare rsFC among the three groups. Statistical significance was set at corrected *p* < 0.05 (uncorrected *p* < 0.01) using Gaussian random field correction, which we performed using the DPABI software. We extracted *Z* values from the gray matter mask showing significant differences among the three groups. Post hoc, we performed two-sample *t* tests of the *Z* values between each pair of groups (HC vs. female patients with MDD with suicidal ideation, HC vs. female patients with MDD without suicidal ideation, and female patients with MDD with suicidal ideation vs. without suicidal ideation) using SPSS (*p* < 0.05).

## Results

### Demographics and clinical characteristics

Following the Beck 19-item Scale for Suicide Ideation, we identified 15 female participants with suicidal ideation and 24 female participants without suicidal ideation. A comparison of demographic characteristics for each group is shown in Table 1. We did not identify significant differences among the three groups in age or education, but we did observe significant differences in HAMD scores (*p* < 0.001) among the three groups by analysis of one-way ANOVA. Post hoc analyses showed higher HAMD scores in female participants in the MDD with suicidal ideation and MDD without suicidal ideation groups compared with HAMD scores in the HC group (*p* < 0.001); however, we did not observe significant differences in HAMD scores between female participants in the MDD with suicidal ideation and MDD without suicidal ideation groups (*p *= 0.06).

**Table 1:** Demographics and clinical data of female participants

	MDD with suicidal ideation (*n* = 15)	MDD without suicidal ideation (*n* = 24)	HC (*n* = 39)	*F*/*χ*^2^	*p*
Age (years)^a^	32.47 ± 11.39	33.50 ± 6.70	29.15 ± 9.33	1.91	0.16
Education (years)^a^	13.67 ± 2.55	13.54 ± 3.02	13.10 ± 2.60	0.32	0.72
HAMD score^a^	22.40 ± 8.62	17.21 ± 8.97	1.26 ± 2.02	72.58	0.000
Lifetime suicide attempts^b^	3 (20%)	3 (12.5%)	0	0.39*	0.53*

*HC* healthy controls, *HAMD* Hamilton Depression Rating Scale

* Compared between patient groups

^a^Data are presented as mean ± SD (standard deviation)

^b^Data are presented as *n* (%)

### Group differences in amygdala rsFC

**Fig. 1:**
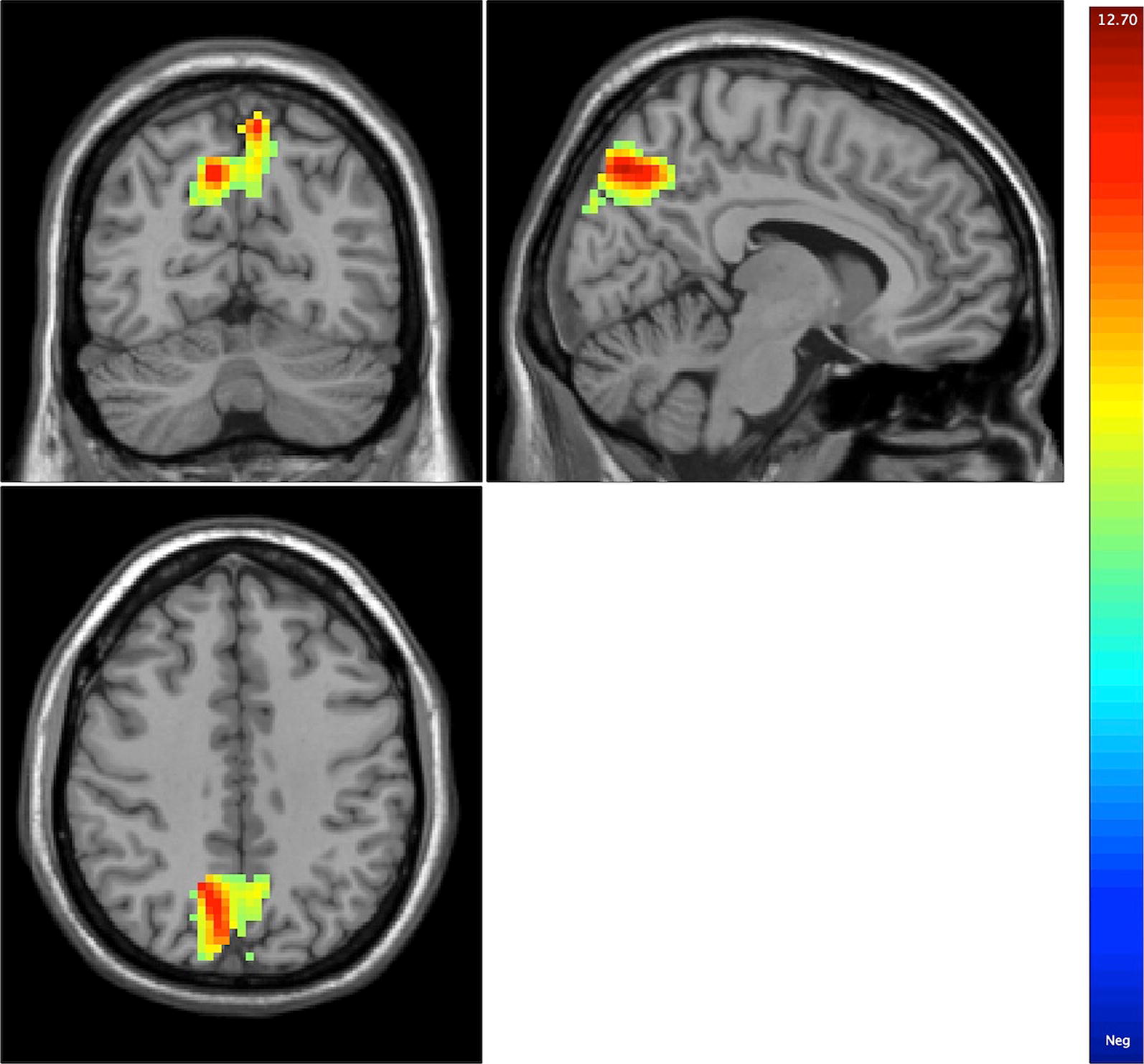
Results of one-way ANOVA showing abnormalities in amygdala–precuneus/cuneus resting-state functional connectivity in a comparison of the three groups

The results of one-way ANOVA showed that there were significant differences in amygdala–precuneus/cuneus rsFC in a comparison of the three groups (*p* < 0.05, corrected). Brain regions showed significant changes in functional connectivity from bilateral amygdala to bilateral precuneus/cuneus (MNI coordinates for the maximal point of difference: *x* = − 6 mm, *y* = − 63 mm, *z* = 63 mm, 628 voxels, *T* = 11.57, *p* < 0.05, corrected; Fig. 1).

**Fig. 2:**
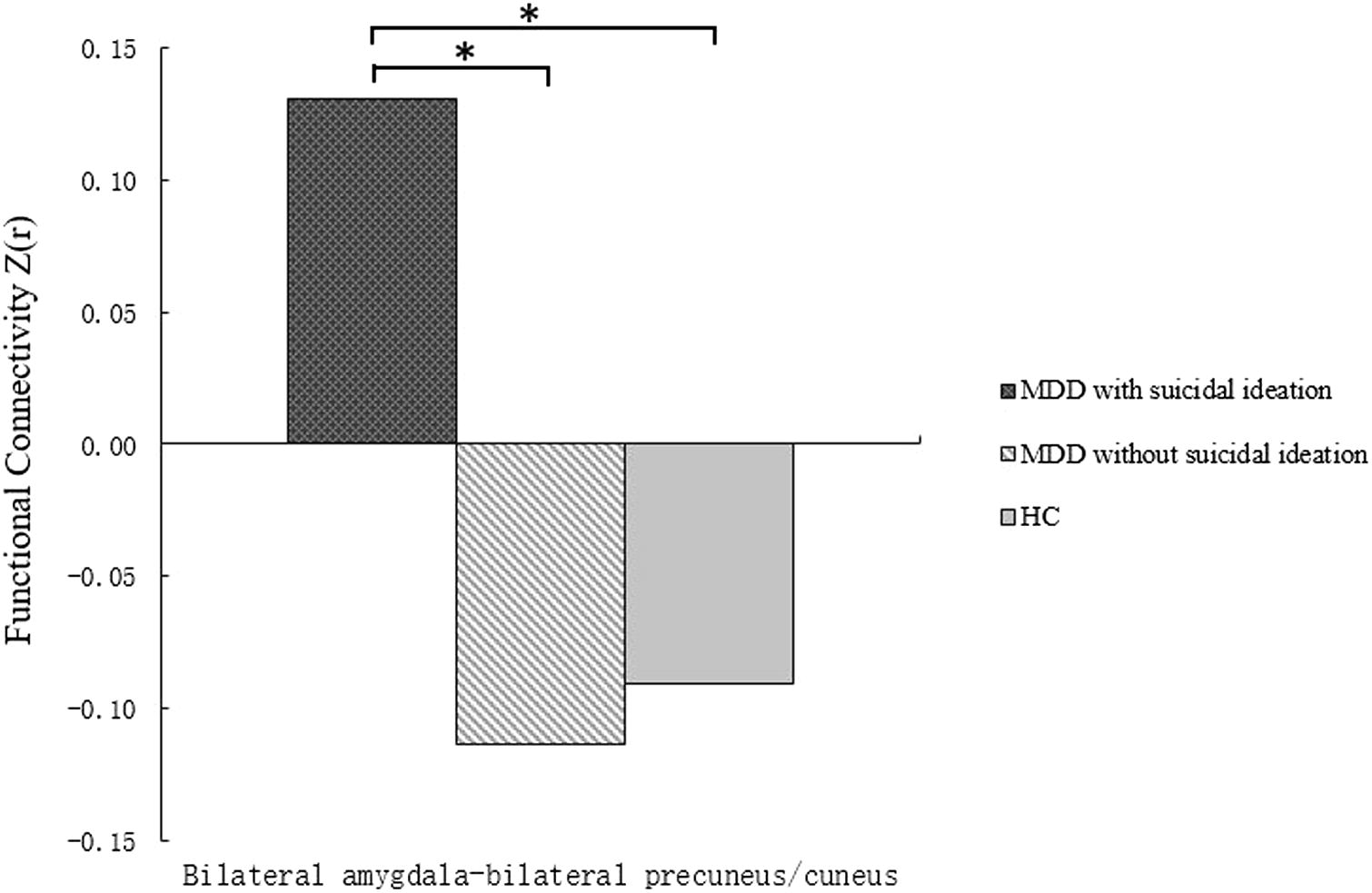
Post hoc comparison showing *Z* value differences at average voxel between each pair group (HC vs. MDD with suicidal ideation, HC vs. MDD without suicidal ideation, and MDD with suicidal ideation vs. MDD without suicidal ideation), *p < 0.01. *HC* healthy control

Female patients with first-episode MDD with suicidal ideation showed greater (positive) amygdala–precuneus/cuneus rsFC in contrast to negative amygdala precuneus rsFC in female patients with first-episode MDD without suicidal ideation and in HC participants. Post hoc pairwise comparisons indicated that the amygdala–precuneus/cuneus rsFC were increased significantly in female patients with first-episode MDD with suicidal ideation separately compared with female patients with first-episode MDD without suicidal ideation or the HC group (*p* < 0.05, corrected). We did not observe significant differences in amygdala–precuneus/cuneus rsFC between female patients with first-episode MDD without suicidal ideation and the HC group (Fig. 2).

### Clinical variables

Exploratory analyses did not reveal any significant correlations between rsFC in regions showing significant group differences and scores on HAMD in women with first-episode MDD with suicidal ideation and women with first-episode MDD without suicidal ideation.

## Discussion

In this study, depression severity in patients groups was higher than health control group. There may be deeply related with the disease of MDD itself. However, our results suggested that the depression severity was not significantly different between two patients groups, which is consistent with the another study of brain structures in suicidal and nonsuicidal female patients with MDD [24]. In our study, we reported that changes in amygdala–precuneus/cuneus rsFC in female patients with first-episode MDD with suicidal ideation were different from female patients with first-episode MDD without suicidal ideation and HC group. However, there were not significant differences in amygdala–precuneus/cuneus rsFC between patients with first-episode MDD without suicidal ideation and the HC group. These findings also suggest that the mechanism underlying suicidal ideation may be related to an abnormality in amygdala–precuneus/cuneus neural circuitry in female patients with first-episode MDD.

Interestingly, two key brain regions underlying suicidal ideation, the amygdala and precuneus, have been strongly implicated in female patients with first-episode MDD. As stated in preface, the amygdala may be a brain region related to the mechanisms of suicidal behavior with MDD. The previous study reported that impulsivity in patients with MDD with a history of suicide attempts was associated with an altered paralimbic (precuneus) encoding of value differences during intertemporal choice [9]. A study of the structural brain found that, among suicide attempters with psychotic disorders, a history of high-lethality attempts was associated with significantly smaller volumes of gray matter in the right cuneus [39]. Studies of fMRI showed that past suicidal behavior in people with the early course psychotic MDD was associated with lower activity in midline parietal regions, including the cuneus and precuneus, when performing cognitive control tasks [40], and the correlation between BOLD signal and relief was greater in nonsuicidal, self-injury patients in areas associated with reward or pain and addiction, including the anterior precuneus [41]. Why precuneus/cuneus may be related to suicide due to its own functions. The previous fMRI studies showed that the precuneus may be related significantly to emotion processing [42–45]. For example, one study of neural correlates of intentional and incidental self-processing suggested that self-processing involves distinct processes and can occur in areas, including the left precuneus, previously implicated in self-awareness [42]. The same regions including the precuneus constitute a functional network of reflective self-awareness that is thought to be a core function of consciousness [43]. The precuneus is associated with mentalizing, self-reference, and autobiographic information [44]. A review of the precuneus functional anatomy and behavioral correlates showed that recent functional imaging findings in healthy subjects suggest a central role for the precuneus in a wide spectrum of highly integrated tasks, including visuospatial imagery, episodic memory retrieval, and self-processing operations—namely, first-person perspective taking and an experience of agency [45]. Therefore, we hypothesize that the precuneus/cuneus may be also associated with the patients with MDD with suicidal behavior.

The circuit of the amygdala–precuneus has been highlighted in MDD, because a recent study reported that adolescents with MDD had positive rsFC between the amygdala and precuneus in contrast to healthy adolescents who showed negative rsFC in this circuit [28], whether this circuit is related to suicide is unclear in this study. From the above studies, those findings do confirm our results that an abnormality in the neural circuitry of the amygdala–precuneus/cuneus may be an important mechanism in suicidal ideation in patients with first-episode MDD. We speculated that the functional connectivity between the amygdala and precuneus could be very important to MDD with suicidal behavior. Thus far, no detailed studies of gender differences have been conducted in amygdala–precuneus/cuneus functional connectivity in MDD with suicidal behavior. Therefore, our results need to be proved by future research. The present study provided evidence that the abnormalities in amygdala–precuneus/cuneus functional connectivity might present the trait feature for female in MDD with suicidal ideation. Moreover, they may indicate potential differentiating markers may improve the early prevention in female in MDD with suicidal ideation. In addition, the clinicians could treat patients in MDD with suicidal ideation using physical therapy-related brain regions. The first limitation in this study is that the assessments of Axis I disorders were performed according to the DSM-IV, not the DSM-V, which could affect the accuracy of the diagnoses. Then, we only investigated the abnormalities of neural circuitry in females with MDD, and did not study males with MDD. Another limitation is the small sample size of the MDD with suicidal ideation group.

## Conclusions

Our findings suggest that suicidal ideation in female patients with first-episode MDD may be related to an abnormality in the amygdala neural circuitry. The abnormality in amygdala–precuneus/cuneus functional connectivity might present the trait feature for suicide in women with first-episode MDD. The precuneus/cuneus may be an important region related to suicide and thus requires future study.

## Abbreviations

MDD: major depressive disorder
HC: healthy controls
fMRI: functional magnetic resonance imaging
rsFC: resting-state functional connectivity
SCID: the Structured Clinical Interview for DSM-IV Axis I Disorders
HAMD: the Hamilton Depression Rating Scale
